# Supplementary material for: Ring Finger Protein 125 Is an Anti-Proliferative Tumor Suppressor in Hepatocellular Carcinoma
Source: Cancers (Basel). 2022 May 24;14(11):2589. doi: 10.3390/cancers14112589 (PMC9179258; doi:10.3390/cancers14112589)
Supplement: Supplementary file 1 [file cancers-14-02589-s001.zip › cancers-1700623-Supplementary Figures.pdf]

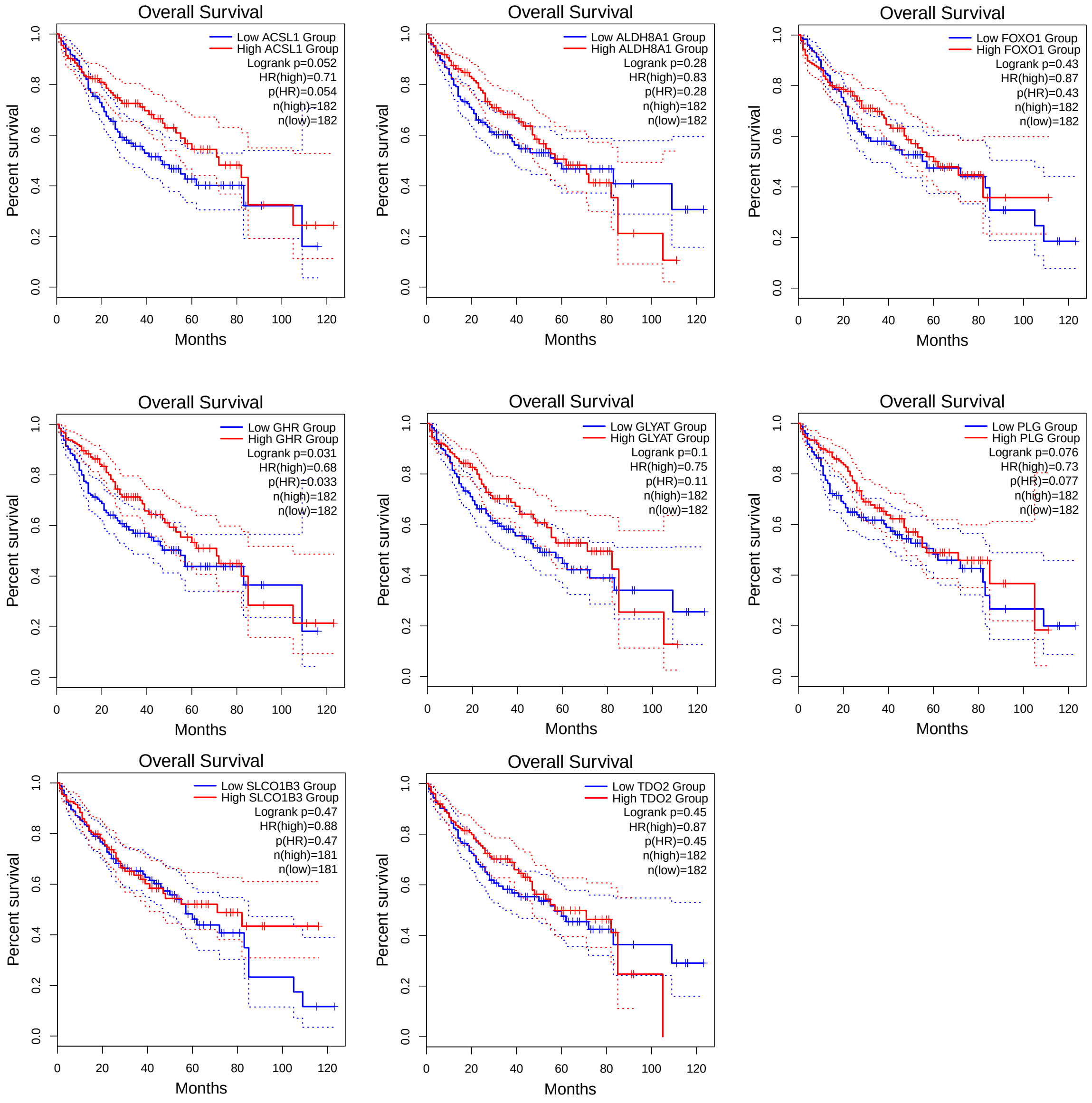

Figure S1. **Kaplan-Mayer curves of overall survival in 364 HCC patients in the TCGA dataset stratified by expression levels of 8 candidate cancer genes**.

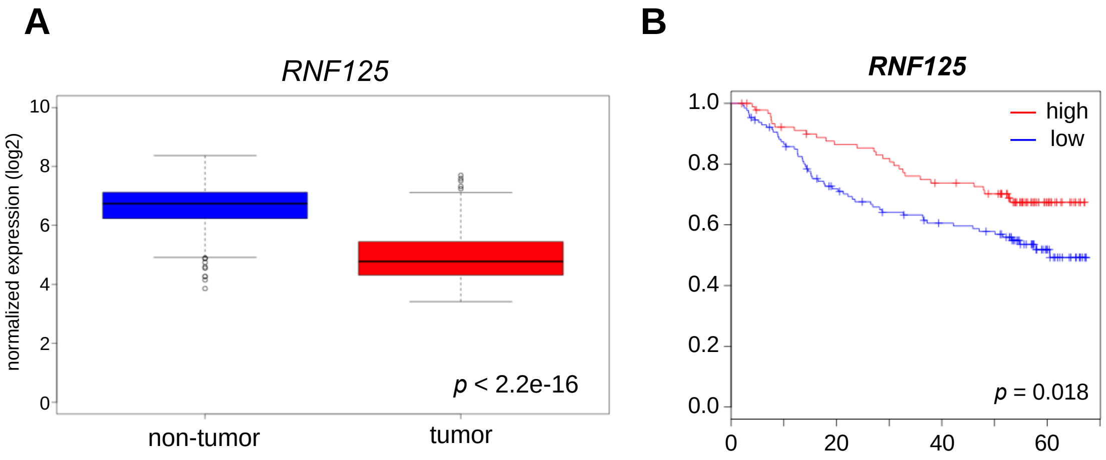

Figure S2. (A) Relative gene expression levels of *RNF125* in tumor and non-tumor tissue of 221 human HCC patients (GSE14520). **(B)** Kaplan-Mayer curves of overall survival in 221 HCC patients (GSE14520) stratified by *RNF125* expression levels.

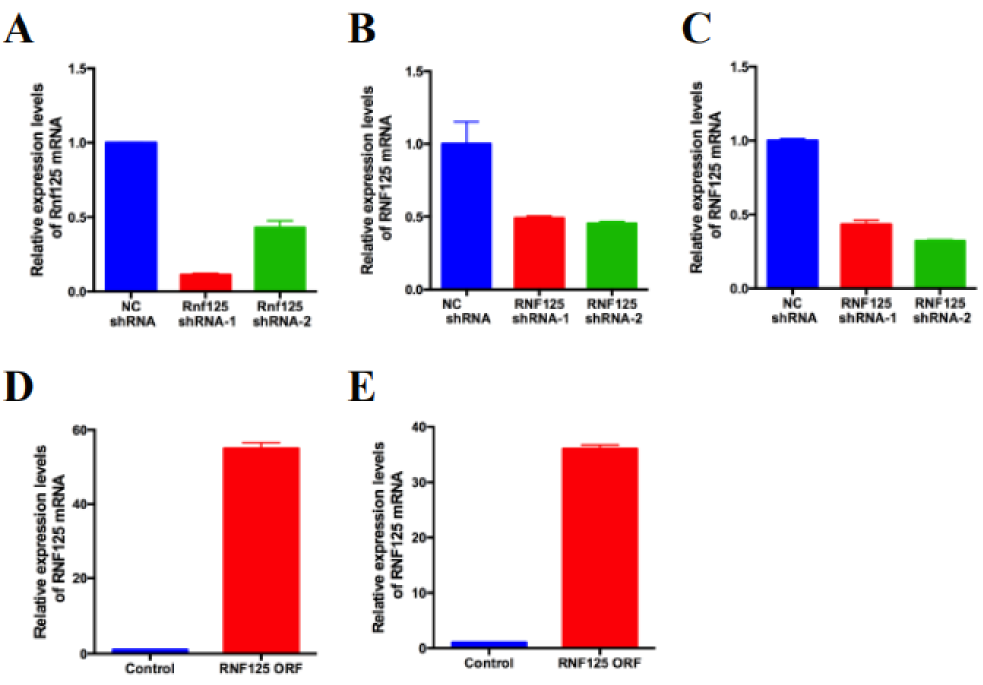

Figure S3. RNF125 knockdown and overexpression in HCC cells.

(**A**) Knockdown of Rnf125 in mouse immortalized liver progenitor cells (LPCs). (**B**) Knockdown of RNF125 in HepG2 HCC cells. (**C**) Knockdown of RNF125 in SNU-398 HCC cells. (**D**) Overexpression of RNF125 in PLC/PRF/5 HCC cells. (**E**) Overexpression of RNF125 in HepB3 HCC cells.

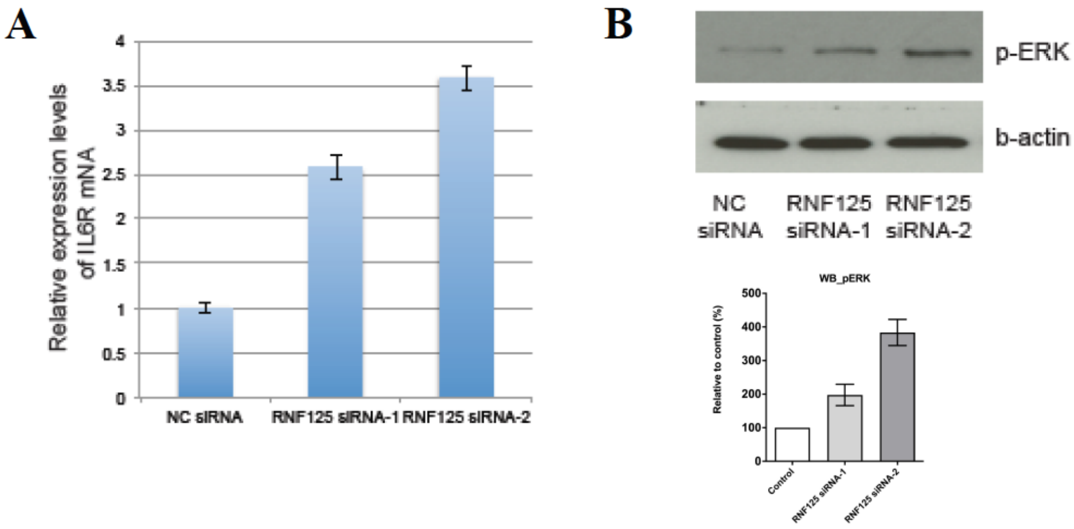

Figure S4. RNF125 knockdown upregulates IL6R and phosphorylated ERK.

(**A**) RT-qPCR results showed upregulation of IL6R after RNF125 knockdown (* $p < 0.05$). (**B**) Knockdown of RNF125 led to increased level of phosphorylated ERK (* $p < 0.05$).
